# A TRIP230-Retinoblastoma Protein Complex Regulates Hypoxia-Inducible Factor-1α-Mediated Transcription and Cancer Cell Invasion

**DOI:** 10.1371/journal.pone.0099214

**Published:** 2014-06-11

**Authors:** Mark P. Labrecque, Mandeep K. Takhar, Julienne M. Jagdeo, Kevin J. Tam, Christina Chiu, Te-Yu Wang, Gratien G. Prefontaine, Michael E. Cox, Timothy V. Beischlag

**Affiliations:** 1 Faculty of Health Sciences, Simon Fraser University, Burnaby, British Columbia, Canada; 2 Department of Urologic Sciences, The Vancouver Prostate Center, University of British Columbia, Vancouver, British Columbia, Canada; University of Dundee, United Kingdom

## Abstract

Localized hypoxia in solid tumors activates transcriptional programs that promote the metastatic transformation of cells. Like hypoxia-inducible hyper-vascularization, loss of the retinoblastoma protein (Rb) is a trait common to advanced stages of tumor progression in many metastatic cancers. However, no link between the role of Rb and hypoxia-driven metastatic processes has been established. We demonstrated that Rb is a key mediator of the hypoxic response mediated by HIF1α/β, the master regulator of the hypoxia response, and its essential co-activator, the thyroid hormone receptor/retinoblastoma-interacting protein (TRIP230). Furthermore, loss of Rb unmasks the full co-activation potential of TRIP230. Using small inhibitory RNA approaches *in vivo*, we established that Rb attenuates the normal physiological response to hypoxia by HIF1α. Notably, loss of Rb results in hypoxia-dependent biochemical changes that promote acquisition of an invasive phenotype in MCF7 breast cancer cells. In addition, Rb is present in HIF1α-ARNT/HIF1β transcriptional complexes associated with TRIP230 as determined by co-immuno-precipitation, GST-pull-down and ChIP assays. These results demonstrate that Rb is a negative modulator of hypoxia-regulated transcription by virtue of its direct effects on the HIF1 complex. This work represents the first link between the functional ablation of Rb in tumor cells and HIF1α-dependent transcriptional activation and invasion.

## Introduction

Hypoxia inducible factor-1α (HIF1α), its orthologue HIF2α, and their dimerization partner the aryl hydrocarbon receptor nuclear translocator, (ARNT or HIF1β) which make up the HIF1 complex [1], [2] regulates a cell’s response to conditions of low oxygen. In healthy tissue, the HIF1 complex directs the ordered and tightly regulated expression of genes controlling the *de novo* synthesis of new vasculature to support tissue growth or tissue re-perfusion. During hypoxia, HIF1α accumulates, translocates to the nucleus, and binds ARNT. The HIF1 complex recruits co-activators including CBP/p300 [3], and Brm/Brg-1[4] and activates the expression of genes, such as vascular endothelial growth factor (VEGF), erythropoietin (EPO) and the metastatic markers, CXCR4 and procollagen lysyl hydroxylase 2 (PLOD2) [1], [5], [6]. Evidence suggests that the HIF1 complex can activate gene expression independently or in concert with other transcription factors [7], [8]. Demonstration that HIF1α is capable of interacting with c-Myc, Notch and more recently FOXA2 to direct ordered transcription and enhance tumor formation [9]–[11] leaves open the possibility that the HIF1 complex is a core transcriptional unit that modulates multiple intracellular signaling networks, many of which may be involved in metastatic transformation. Thus, many of the molecules that control different aspects of HIF1 function have yet to be identified.

The HIF1 complex carries out this function by recruiting transcriptional co-activator proteins including the thyroid hormone receptor/retinoblastoma-interacting protein-230 (TRIP230) to the regulatory regions of hypoxia-responsive genes to activate transcription [12]. TRIP230, was initially identified as a thyroid hormone receptor (TR)-interacting protein that enhanced TRs activity [13]. In addition, TRIP230 has been isolated as part of the p160 co-activator complex [14], a bona fide ARNT co-activator complex [15]. Importantly, we have demonstrated that TRIP230 is recruited by ARNT as a transcriptional co-activator and it is essential for the transcriptional activity of the HIF1 complex [12]. Furthermore, it was shown that TRIP230 interacts with Rb and that Rb attenuates TRIP230-enhanced TR-driven transcription [16]. This and a subsequent study demonstrated that only the hyper-phosphorylated form of Rb interacts with TRIP230 [17] highlighting a function for Rb distinct from its canonical E2F-dependent regulation of cell cycle, specific to its hypo-phosphorylated form.

Loss of *RB1*, the gene that codes for Rb [18], and or loss-of-function of Rb is associated with the development and metastatic progression of many other solid tumors including cancers of the ovary, lung, breast, prostate and brain [19]–[23]. The best understood function of Rb is that of cell cycle regulator repressing E2F transcription factor function thereby mediating cell proliferation and differentiation [24]. Hypo-phosphorylated Rb blocks cell cycle progression by binding to E2F transcription factors and affecting E2F-dependent transcriptional outcomes. It does so by recruiting chromatin-remodeling transcriptional repressor proteins such as Sin3a/b, HDACs, SUV39H1 and DNMT1 [25]–[27]. Hyper-phosphorylated Rb fails to repress E2Fs and allows them to activate or repress various gene expression programs [24].

Recent studies suggest that Rb may have physiological roles in addition to its canonical E2F function [28]. Previously, we demonstrated a direct interaction between TRIP230 and ARNT [12]. In addition, we demonstrated that TRIP230 was indispensable for transcription mediated by two distinct dimerization partners of ARNT, namely the aryl hydrocarbon receptor and HIF1α [12]. In this report, we provide the first evidence for the existence of an Rb-TRIP230-ARNT complex that mediates HIF1 transcription. In addition, we demonstrate that Rb attenuates the activity of ARNT transcriptional complexes by virtue of its association with TRIP230 and independent of E2F. Ultimately, this work reveals the ability of Rb to modulate HIF1-activated gene expression with consequences for cancer cell transformation.

## Results

### HIF1-regulated Gene Expression is Enhanced by siRNA Knock-down of Rb

**Figure 1:**
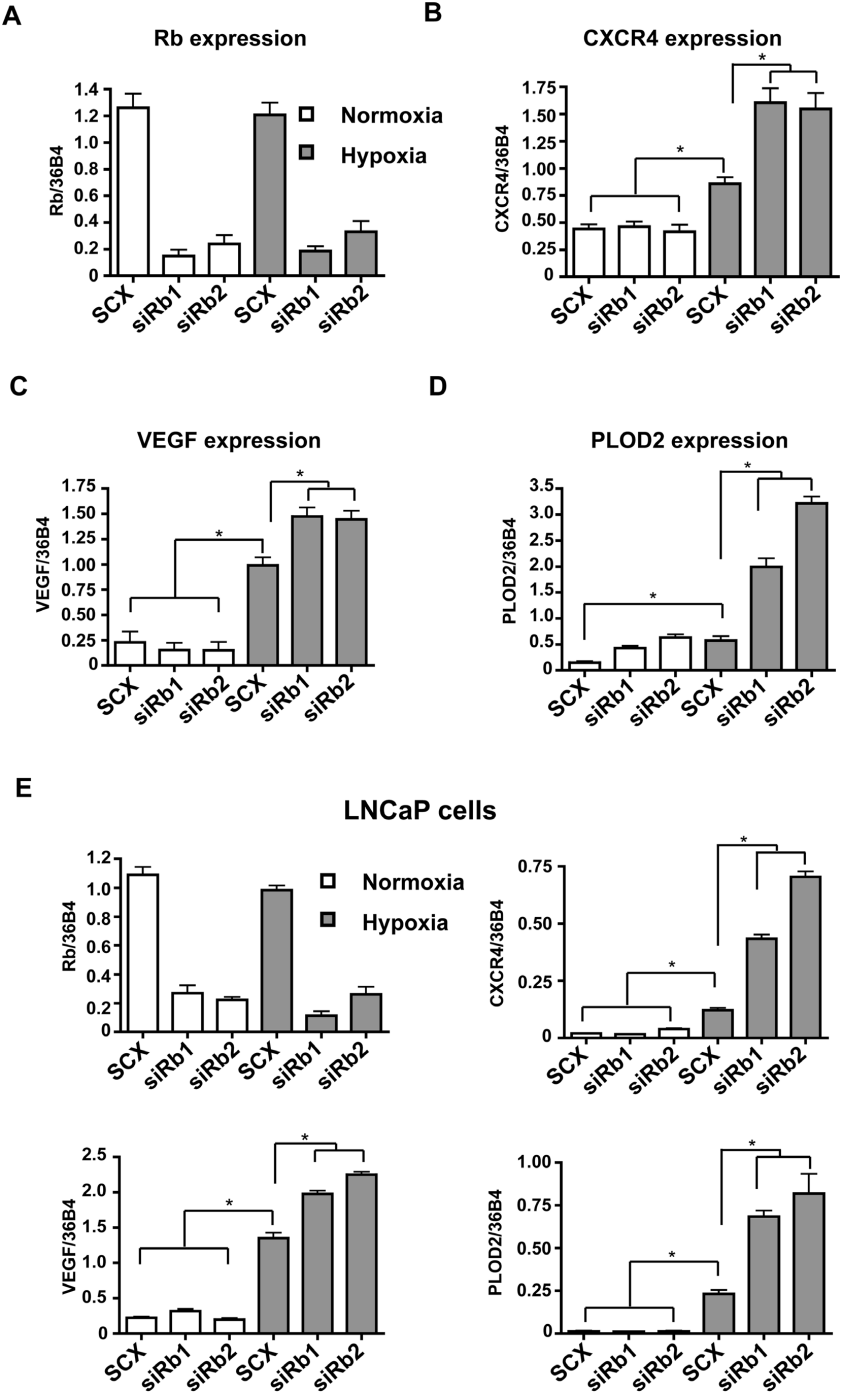
Rb represses HIF1-regulated target gene mRNA in MCF7 human breast cancer cells and LNCaP human prostate cancer cells. MCF7 cells (**A, B, C and D**) and LNCaP cells (**E**) were transfected with either scrambled siRNA (SCX) or Rb siRNAs siRb 1, and siRb 2. Twenty-four hours after transfection cells were either maintained under normoxic conditions or 1% O_2_ for a further 24 h. Gene expression was determined by quantitative real-time PCR after isolation and reverse transcription of total RNA. VEGF, CXCR4, PLOD2 and Rb expression were normalized to constitutively active 36B4 gene expression. Open bars represent normoxia (20% O_2_) and closed (grey) bars represent hypoxia (1% O_2_). Error bars represent ± S.D. *p<0.05.

TRIP230 is known to bind directly to TR and ARNT [12], [16]. Given that hyper-phosphorylated-Rb represses TRIP230 co-activated TR activity [16], [17] and that TRIP230 expression is required for transcriptional activity of the ARNT partners, AHR and HIF1α [12], we hypothesized that Rb might attenuate hypoxia-inducible gene transcription. In order to examine the role of Rb on the accumulation of hypoxia-inducible target gene mRNA species, we depleted Rb in human cancer cell lines by siRNA-mediated gene *knock-down*. MCF7 and LNCaP cells were transfected with either scrambled (SCX) control siRNA or two Rb-specific siRNAs and mRNA accumulation of HIF1 target genes exposed to normoxia and hypoxia were compared (Figure 1). Rb RNA and protein levels were equivalently suppressed under both normoxic and hypoxic conditions (Figures 1A, and E). No change was observed in CXCR4 or VEGF expression in Rb siRNA transfected cells under normoxic conditions, however an increase in PLOD2 mRNA accumulation under normoxic conditions was observed in MCF7 cells (Figure 1D) but not in LNCaP cells (Figure 1E). In addition, MCF7 cells transfected with the Rb-specific siRNAs exhibited significantly increased mRNA accumulation of the HIF1 target genes CXCR4, VEGF and PLOD2 under hypoxic conditions (Figure 1B–D). A similar hypoxia-dependent effect on CXCR4, VEGF and PLOD2 induction was observed in response to suppressed Rb expression in LNCaP prostate cancer cells (Figure 1E) suggesting that this observation is not cell type specific.

### Rb and Rb-associated Repressor Proteins are Recruited to the Regulatory Regions of Hypoxia Inducible Genes during Activated Transcription

**Figure 2:**
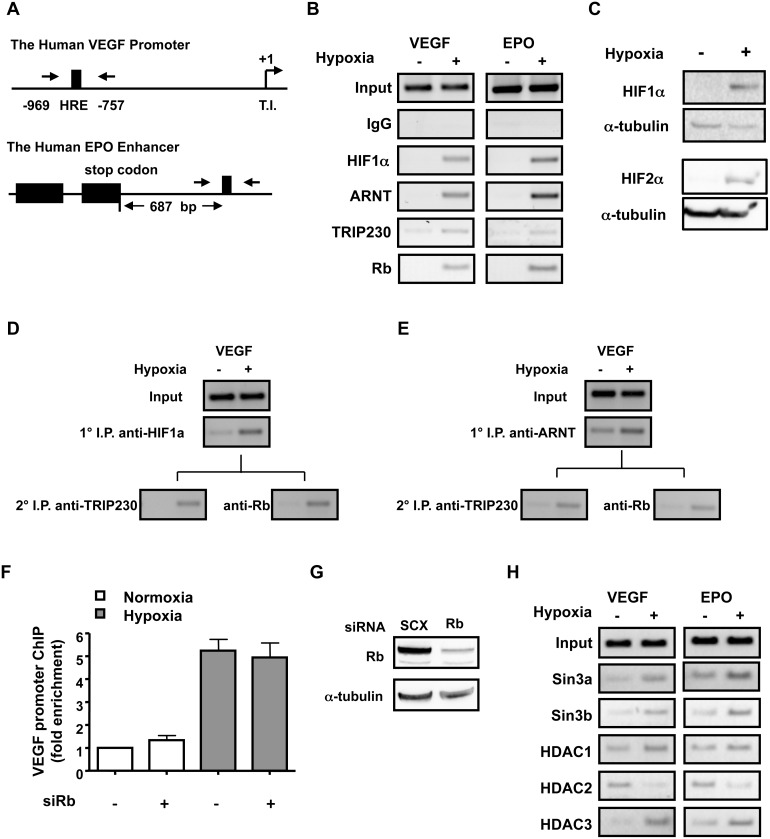
HIF1α, ARNT, TRIP230, Rb and Rb-associated repressor proteins occupy hypoxia responsive regulatory regions of HIF1-regulated genes in a hypoxia-dependent fashion. (**A**) A schematic of the VEGF proximal promoter and EPO enhancer and the relative location of oligonucleotides used for PCR amplification. (**B**) The status of HIF1α, ARNT, TRIP230, and Rb at the VEGF promoter and EPO enhancer in MCF7 cells as assayed by chromatin-immuno-precipitation (ChIP) and polymerase chain reaction (PCR) and compared to amplification reactions derived from lysates precipitated with control IgG. All ChIPs were performed at least three times except where indicated. (**C**) HIF1α and HIF2α protein levels are dramatically enriched during hypoxia in MCF7 cells. MCF7 cells were maintained in culture in either 20% or 1% O_2_ for 24 h. Nuclear extracts were analyzed by immuno-blot and membranes were probed with affinity-purified antibodies to HIF1α and α-tubulin. Sequential ChIP of the proximal VEGF promoter and EPO enhancer using either anti-HIF1α (**D**) or anti-ARNT (**E**) affinity purified antibodies followed by immuno-precipitation with anti-TRIP230 and anti-Rb antibodies. (**F**) ChIP of the VEGF promoter after in MCF7 cells after transfection with either scrambled siRNA or siRb1 and immuno-precipitation with anti-TRIP230 antibody. Values are expressed as fold enrichment over control and were determined by quantitative real-time PCR. Each experimental value was corrected for input and experiments were performed twice. (**G**) Immuno-blot analysis of siRNA-transfected MCF7 cell lysates used in ChIP experiments. (**H**) ChIP of Rb-repressor complex proteins in MCF7 cells. Cells were treated as described above and chromatin complexes were isolated with affinity-purified antibodies directed to Sin3a, Sin3b, and HDACs 1–3.

We observed that loss of Rb resulted in exaggerated expression of HIF1 target genes in a hypoxia-dependent fashion. Thus, we were interested to determine if the presence of Rb could be recorded over the regulatory regions HIF1-regulated genes harboring well characterized hypoxia response elements during activated transcription; namely the VEGF promoter and EPO enhancer. A schematic of these regions and the placement of oligonucleotides for PCR amplification of chromatin are depicted in Figure 2A. Chromatin immuno-precipitation (ChIP) analysis revealed that Rb associates with regulatory regions of the HIF1-regulated genes, VEGF and EPO in a hypoxia-dependent fashion in MCF7 cells (Figure 2B). To ensure that our experimental conditions produced the appropriate response to hypoxia, we also precipitated chromatin with antibodies to HIF1α, ARNT, TRIP230 or a control rabbit IgG. Control IgG was incapable of precipitating chromatin containing the VEGF and EPO regulatory regions. As we have demonstrated previously [12], we were more readily able to amplify chromatin precipitated from hypoxia treated lysates than from those derived from lysates maintained under normoxic conditions using the HIF1α, ARNT, TRIP230 and Rb antibodies. We could amplify low levels of the VEGF promoter and EPO enhancer under normoxic conditions (Figure 2B) when precipitating chromatin with our TRIP230 antibody, therefore, we cannot discount the possibility that TRIP230 associates with HREs at low levels despite normal oxygen tension. However, our inability to detect HIF1α or HIF2α protein under these conditions (Figure 2C) suggests the possibility that Rb is not recruited to these HRE regions under normoxic conditions.

We expanded our investigations in an attempt to determine if TRIP230 and Rb have the ability to interact with individual DNA elements in concert with HIF1α and ARNT at hypoxic response elements. We performed a sequential two-step ChIP assay, first isolating chromatin with affinity-purified antibodies to ARNT or HIF1α followed by precipitation of these purified chromatin fractions with antibodies to TRIP230 and Rb. In each case VEGF promoter DNA was amplified by PCR in a hypoxia-dependent fashion (Figure 2D and E) strongly suggesting that HIF1α, ARNT, TRIP230 and Rb are present at HREs in a multi-protein complex. In addition, knock-down of Rb as assessed by immuno-blot (Figure 2G), did not result in further enrichment of TRIP230 at the VEGF promoter (Figure 2F) suggesting that Rb does not interfere with TRIP230 recruitment to HIF1 responsive elements.

Finally, we were interested to see if known Rb-associated repressor complexes [26], [27] were present at these elements during hypoxia-driven transcription. ChIP analysis revealed the Sin3a/b, HDAC1 and HDAC3 were enriched at the HIF-responsive regulatory regions of both the VEGF and EPO genes under hypoxic conditions (Figure 2H) supporting our hypothesis that Rb is part of transcriptional repressor complex acting on HIF1-regulated transcription. In contrast, HDAC2 behaved in a more canonical fashion and was dismissed from these regions in a hypoxia-dependent fashion (Figure 2H). These data suggest that transcriptional repressor proteins are recruited to hypoxia-regulated genes during activated transcription. Furthermore, these observations support the hypothesis that transcription must be attenuated to ensure the appropriate transcriptional response.

### ARNT and TRIP230 are Essential for Rb-regulation of HIF1 Activity

**Figure 3:**
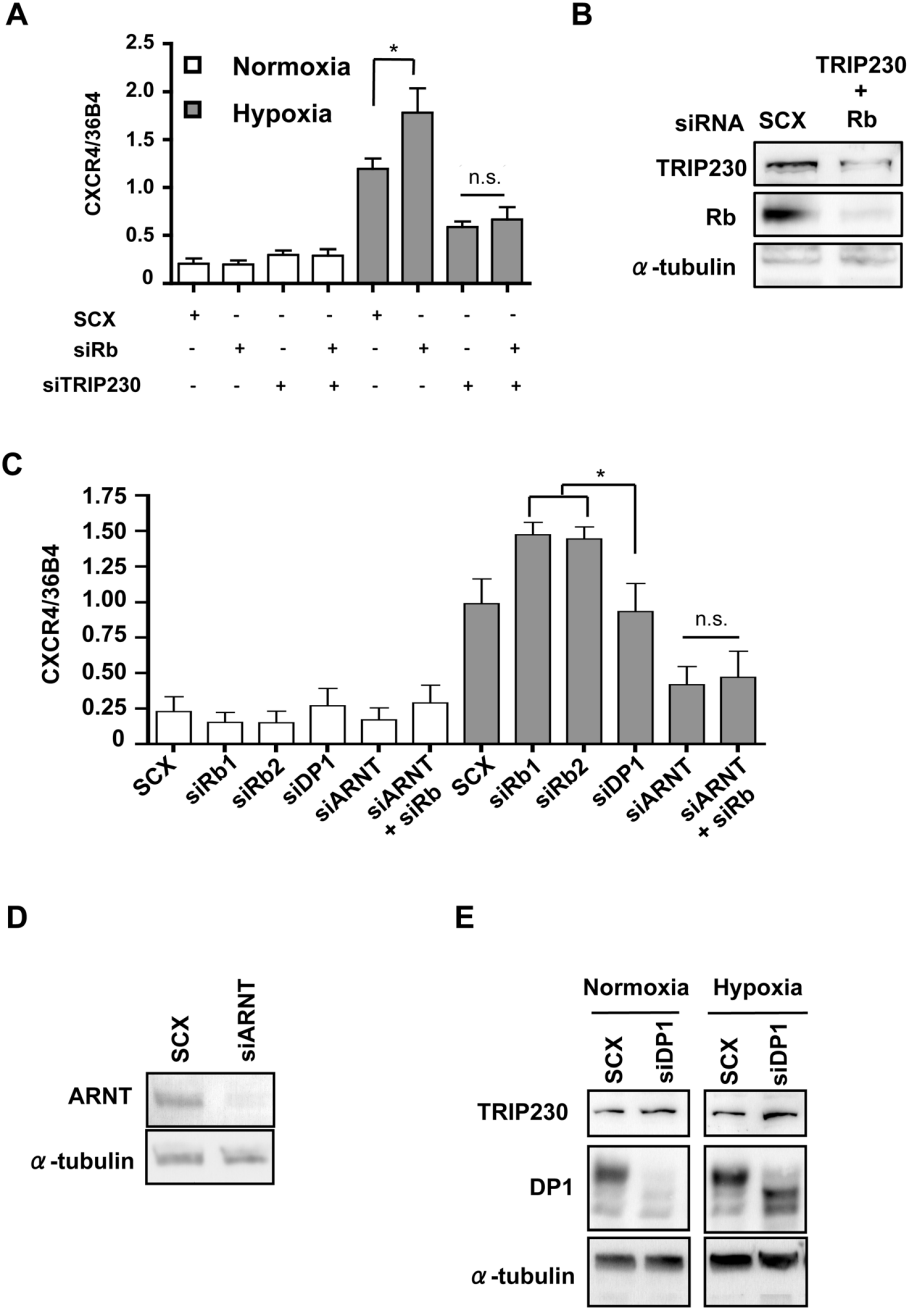
ARNT and TRIP230 are essential for Rb-regulation of HIF1. (**A**) MCF7 cells were transfected with either scrambled siRNA (SCX) or Rb siRNAs siRb 1, and siRb 2, or a combination of TRIP230 siRNA and siRb1. Twenty-four hours after transfection cells were either maintained under normoxic conditions or 1% O_2_ for a further 24 h. Gene expression was determined by quantitative real-time PCR after isolation and reverse transcription of total RNA. CXCR4 expression was normalized to constitutively active 36B4 gene expression. (**B**) Immuno-blot analysis of TRIP230 and Rb after siRNA transfection with either scrambled siRNA, or a combination of siTRIP230 and siRb. Alpha-tubulin (α-tubulin) was used as a loading control. (**C**) MCF7 cells were transfected with either scrambled siRNA (SCX) or Rb siRNAs siRb 1, and siRb 2, or ARNT, or DP1 siRNA or a combination of ARNT/Rb1 siRNA and treated as described above. (**D**) Immuno-blot of ARNT and α-TUB after transfection with either scrambled control of siRNA directed to ARNT. (**E**) Immuno-blot of DP1, TRIP230 and α-tubulin (α-tubulin). MCF7 cells were transfected with either scrambled control (SCX) or siRNA directed to DP1. Data in figure 3A and 3C were analyzed using a two-way-ANOVA. *p<0.01.

Since Rb and TRIP230 are interacting proteins, the quantitative RT-PCR experiments were repeated using a siRNA directed to TRIP230. Hypoxic gene induction of CXCR4 mRNA accumulation was severely impaired upon knock-down of TRIP230 (Figure 3A and B). Knock-down of Rb under these conditions, as evidenced by immuno-blot analysis (Figure 3B) did not result in any significant increase in CXCR4 mRNA, providing further evidence that this effect is TRIP230-dependent. Images of whole immuno-blots can be found in Figure S1. Furthermore, loss of Rb did not lead to an increase in CXCR4 mRNA accumulation in cells ablated for ARNT by siRNA-mediated knock-down further suggesting that the effect mediated by Rb is hypoxia-dependent (Figure 3C and D). In addition, siRNA-mediated suppression of DP1 expression in MCF7 cells responded to hypoxia as readily as control cells (Figure 3C and E) indicating that the modulatory function of Rb on HIF-regulated genes is independent of E2F and uncoupled from Rb’s canonical role as a cell cycle mediator.

### Loss of Rb Leads to an Increase in HIF1 Target Gene Protein Expression

**Figure 4:**
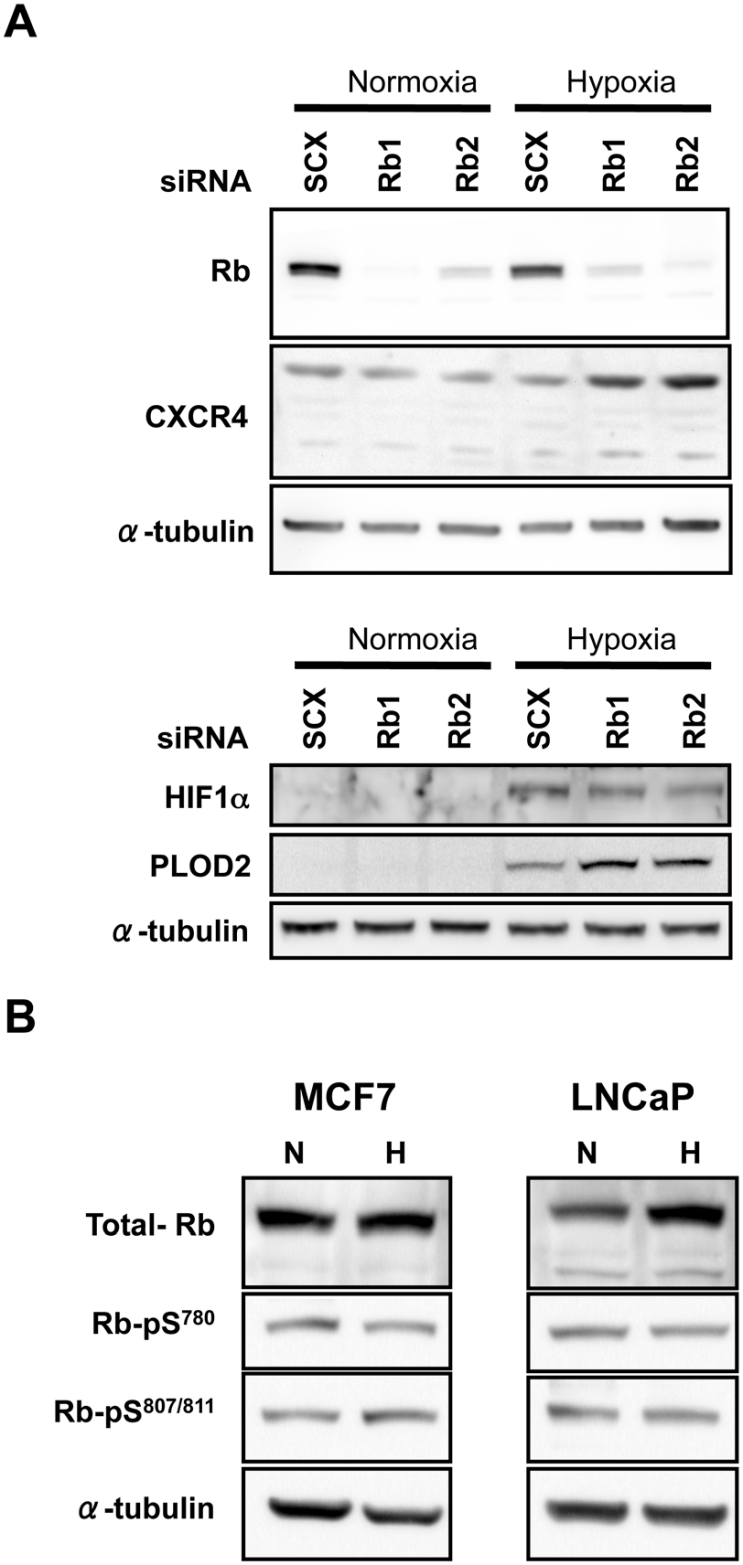
Rb represses HIF1-regulated target gene protein accumulation in MCF7 human breast cancer cells and is phosphorylated at serines 780 and 807/811. MCF7 cells (**A**) were transfected with either scrambled siRNA (SCX) or Rb siRNAs siRb 1, and siRb 2. Rb, CXCR4, HIF1α, PLOD2 and α-tubulin protein levels were assessed by immuno-blot after exposure to atmospheric O_2_ or 1% O_2_ for 48 (Rb and CXCR4) or 96 h (HIF1α, and PLOD2). (**B**) Immuno-blots of whole cell lysates from MCF7 and LNCaP cells either left at normoxia (N) or treated with 1% O_2_ for 6 h (H). Blots were probed with primary antibodies to total Rb, Rb-phospho-serine^780^ (Rb-pS^780^), Rb-phospho-serine^807/811^ (Rb-pS^807/811^), or α-tubulin as a loading control.

We were interested to determine if the effect of Rb-loss on mRNA accumulation was reflected at the protein level of HIF1 target genes and, in particular if pro-metastatic factors were affected. Thus, we also examined the role of Rb in the expression of downstream metastatic markers that are sensitive to hypoxia. Loss of Rb resulted in a concomitant increase in CXCR4 protein levels after 48 h of hypoxia and PLOD2 after 96 h of hypoxia (Figure 4A). Furthermore, a 24 h exposure to hypoxia with Rb knock-down also resulted in an increase in the expression of the mesenchymal marker, vimentin (Figure S2A). In addition, loss of Rb did not increase endogenous levels of HIF1α (Figure 4A), suggesting that the observed hypoxic effect was not due to an increase in HIF1α expression or stability. Finally, previous reports demonstrated that TRIP230 associates with hyper-phosphorylated Rb [16], [17]. Immuno-blotting lysates from MCF7 and LNCaP cells with antibodies directed to Rb, Rb-phospho-serine^780^ and Rb-phospho-serine^807/811^ demonstrate that there is a significant amount of phosphorylated Rb in MCF7 and LNCaP cells (Figure 4B).

### Loss of Rb Promotes an Invasive Phenotype in MCF7 Breast Cancer Cells

**Figure 5:**
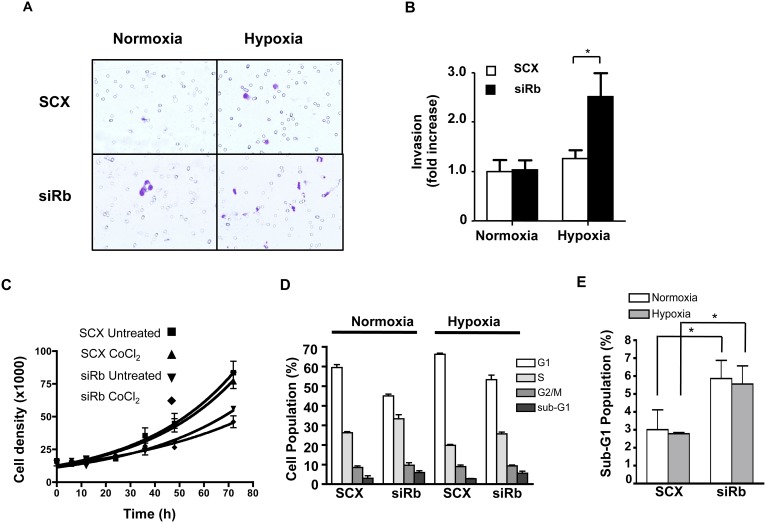
Loss of Rb promotes hypoxia-dependent invasiveness in MCF7 cells in a Matrigel Invasion Assay - MCF7 cells were transfected with scrambled siRNA (SCX) or Rb siRNA as described above. Twenty-four hours after siRNA transfection, the cells were subjected to the Matrigel invasion assay. Plates were incubated in normoxic (20% O_2_) or hypoxic conditions (1% O_2_) at 37°C for 24 h and invading cells were fixed and visualized with toluidine blue. (**A**) Photomicrographs of matrigel-embedded MCF7 cells. (**B**) Numerical representation of relative invasion of matrigel-embedded MCF7 cells after treatment with SCX or siRb and exposure to normoxic or hypoxic conditions (n = 6), (**C**) Knock-down of Rb in MCF7 cells does not alter cell proliferation in response to CoCl_2_. Cells were transfected with siRNA’s as described above. Twenty-four h after transfection, cells were treated with vehicle or 100 µM CoCl_2_ to activate HIF1α and cells were counted at 0, 6, 12, 24, 36 48, and 72 h later. Error bars represent ± S.E.M. *p<0.01.

The ability of Rb knock-down to enhance HIF1 complex transcriptional activity and protein expression led us to assess whether Rb suppression might also enhance hypoxia-induced cell invasion in traditionally non–invasive MCF7 breast cancer cells [29]. For cells with a complete complement of Rb (i.e. cells transfected with SCX control siRNA), hypoxia had no effect on invasion of MCF7 cells into the matrigel (Figure 5A and B). However, siRNA knock-down of Rb led to increased invasion of MCF7 cells under hypoxic conditions but had no effect on invasion under normoxic conditions (Figure 5A and B). These data support the role of Rb as a tumor suppressor of hypoxia-regulated metastatic programs.

We were concerned that the increased invasiveness might be due to a dramatic increase in cell proliferation due to loss of Rb control of the cell cycle. To avoid periods of prolonged, severe hypoxia, we demonstrated that the HIF1α stabilizing agent CoCl_2_ would mimic the effects of hypoxia in the matrigel assay (Figure S2C). These data further support our hypothesis that the observed effects are mediated via the HIF1 complex. With CoCl_2_ established as an effective mimic of hypoxia after knock-down of Rb in the matrigel assay, we were able to determine if the observed increase in cell invasion was due to an increase in cell proliferation (Figure 5C). MCF7 cell growth dynamics were monitored at regular intervals by counting viable cells over a 72 h period. In cells transfected with either SCX control siRNA or Rb siRNA and with separate samples of each maintained either in the presence or absence of CoCl_2_, we observed that loss of Rb decreased proliferation in both un-treated and CoCl_2_-treated cells (Figure 5C) with no significant difference between the other treatments. Likewise, matrigel invasion of SCX control cells was indistinguishable under either condition. Cell sorting after propidium iodide staining revealed a larger proportion of the Rb knock-down cells in the sub-G1 phase of the cell cycle (Figure 5D and E). Taken together, these data strongly suggest that loss of Rb promotes cell invasion in a hypoxia-dependent fashion and that these effects are not due to increased cell number or proliferation.

### Rb Associates with the PAS-B/TRIP230-interaction Domain of the ARNT Protein

**Figure 6:**
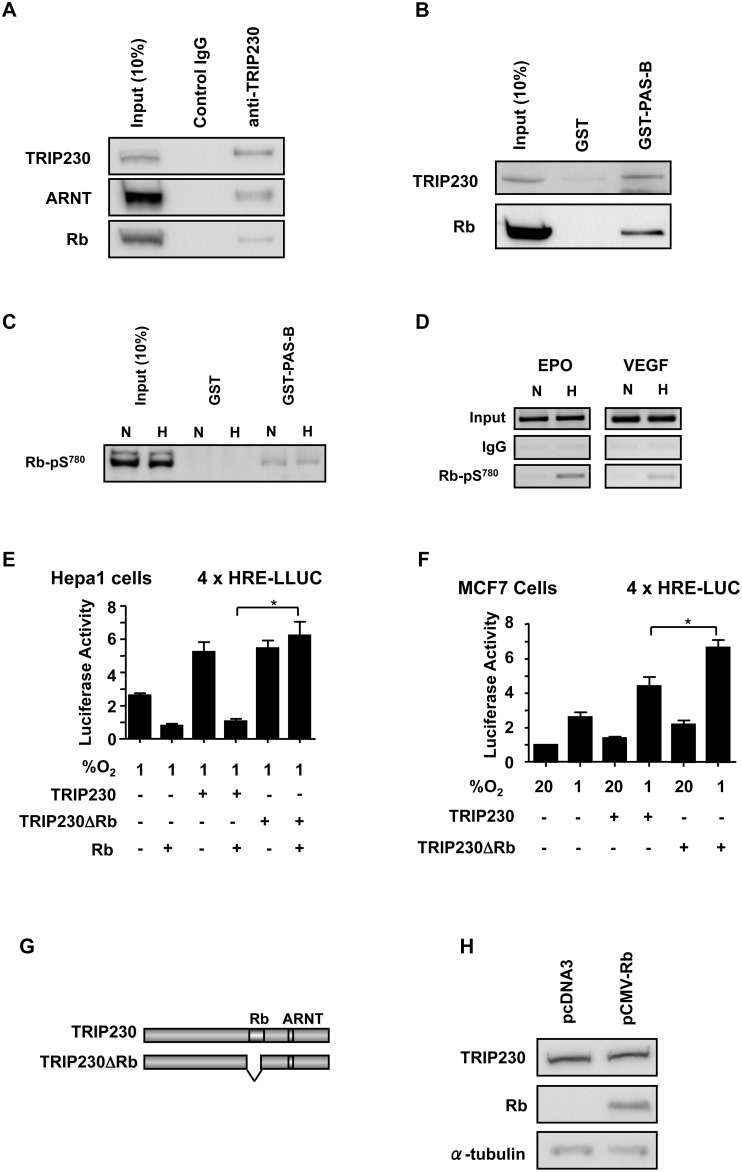
Rb mediates its transcriptional effects on hypoxia-inducible gene regulation through an ARNT-TRIP230-Rb complex. (**A**) Immuno-blot of complexes precipitated using either a mouse monoclonal antibody directed to TRIP230 or mouse control IgG from the nuclear extracts of MCF7 cells. Blots were probed for the presence of TRIP30, ARNT and Rb. The left hand lane of each blot contains nuclear extract representing 10% of input. (**B**) GST-ARNT-PAS-B is capable of pulling down TRIP230 and Rb. Immuno-blot analysis of GST-ARNT-PAS-B pull-down and GST pull-down of TRIP230 and Rb from MCF7 nuclear extracts. GST moieties were fixed to glutathione-agarose beads and incubated for 90 min at 4 degrees C with 500 µg of MCF7 cell nuclear extract. Input lanes were loaded with 250 µg of nuclear extract. Complete blots for panels A and B can be found in Supplemental Figure S1. GST-ARNT-PAS-B is capable of pulling down phosphorylated Rb. (**C**) Immuno-blot analysis of GST-ARNT-PAS-B pull-down and GST pull-down of Rb-phospho-serine^780^ (Rb-pS^780^) from MCF7 nuclear extracts harvested from cells left at normoxia (N) or treated with 1% O_2_ for 6 h (H). (**D**) Chromatin immuno-precipitation assay of EPO enhancer and VEGF promoter regions in MCF7 cells using control or Rb-phospho-serine^780^ antibodies. Cells were treated as described above. Rb attenuates TRIP230-mediated co-activation of ARNT-dependent transcriptional activity. The Rb- and ARNT-interaction domains are indicated. Hepa-1c1c7 cells (**E**) and MCF7 cells (**F**) were transfected with a hypoxia responsive 4xHRE-driven luciferase construct as a reporter, expression plasmids for TRIP230, TRIP230ΔRB and Rb as indicated and subjected to 1% O_2_ or atmospheric (20%) O_2_ for 24 h. Whole cell lysates were assayed for luciferase activity. (**G**) A schematic of the TRIP230ΔRB mutant. (**H**) TRIP230 protein levels are unaffected by transfection of Rb expression plasmid into Hepa1c1c7 cells. Whole cell lysates were analyzed by immuno-blot and membranes were probed with affinity-purified antibodies to TRIP230, Rb and α-tubulin. *p<0.05.

The possibility that ARNT, TRIP230 and Rb could be part of a multimeric complex was explored by immuno-precipitation of TRIP230 associated complexes from the nuclear extracts of MCF7 cells incubated for 6 h under hypoxic conditions (Figure 6A). Immuno-blot analysis revealed ARNT and Rb to be present in the anti-TRIP230 immuno-precipitate while none of the three factors were detected in precipitates of lysates performed with non-immune IgG. These results provide strong evidence that native TRIP230 protein is capable of protein-protein interactions with ARNT and Rb.

Given that previous *in vitro* interaction studies determined that Rb does not directly interact with ARNT [30], we therefore were interested to determine if the TRIP230 interaction domain within ARNT could be used to isolate Rb from MCF7 cell nuclear extracts. Partch and colleagues have identified that the TRIP230 interaction domain within ARNT is located in its PAS-B region [31]. We fused amino acids 344–479 harboring the expanded PAS-B domain of mouse ARNT to GST. Using this minimal interaction domain was done in part to reduce the potential for ARNT to interact with other nuclear proteins. Pull-down of TRIP230 and Rb from nuclear extracts of hypoxia-conditioned MCF7 cells was dramatically enriched using the GST-ARNT-PAS-B domain compared to GST alone (Figure 6B). Thus, it is possible that an ARNT complex including TRIP230 and Rb is formed through the ARNT PAS-B domain. This domain mediates the interaction between ARNT and multiple co-activators that are essential for ARNT-mediated transcription: namely, TRIP230 [12], the p160/NCoA/SRC-family of transcriptional co-activators [15], and CoCoA [32].

Finally, we wished to determine specifically, if hyper-phosphorylated Rb was involved in HIF1-regulated gene transcription. We probed immuno-blots with an anti-phospho-serine^780^ Rb-specific antibody after pull-down using GST-PAS-B. In this fashion, we observed hyper-phosphorylated Rb in blots generated from normoxic and hypoxic MCF7 cell nuclear extracts (Figure 6C). In addition, interrogation of the transcriptional regulatory regions of the HRE-containing VEGF promoter and EPO enhancer revealed the presence of Rb-pSer^780^ in a hypoxia-dependent fashion (Figure 6D).

### TRIP230 Mediates the Repressive Effects of Rb on HIF-regulated Transcription

We have established that Rb co-purifies with TRIP230 and that Rb attenuates the accumulation of hypoxia-inducible target gene mRNA and protein levels. In order to determine if the ability of Rb to modulate HIF1-regulated transcriptional activity was mediated via TRIP230, we examined effects of Rb on the expression of a hypoxia-responsive reporter construct using deletion mutants of TRIP230 in Rb-negative and –positive cell lines. Rb-negative Hepa1c1c7 cells were transfected with an expression plasmid encoding TRIP230, or a transcriptionally competent deletion-mutant of TRIP230 (TRIP230ΔRb; schematic in Figure 6G) lacking the Rb-interaction domain [17], an Rb cDNA expression plasmid, and a synthetic luciferase reporter construct containing a multimerized hypoxia-responsive element (HRE) promoter (Figure 6E). Rb abrogated hypoxic induction of reporter activity in cells transfected with wild-type TRIP230, but was ineffective in cells transfected with the mutant TRIP230. Transfection of Rb into the Hepa1c1c7 cell line had no effect on levels of endogenous TRIP230 protein (Figure 6H). Indeed, titration of increasing amounts of Rb-expression plasmid repressed hypoxia-inducible reporter activity in a dose-dependent manner (Figure S2B). Transfection of the mutant TRIP230 into Rb-positive MCF7 human breast cancer cells further enhanced expression of the reporter gene (Figure 6F), thus acting as a dominant negative likely by competing for HREs with the endogenous wild-type TRIP230. These data collectively suggest that Rb’s attenuating effects on HIF1 transcriptional activity appear to be mediated by TRIP230.

## Discussion

We have determined that Rb attenuates the physiological response to hypoxia by HIF1α and that it is an integral and indispensable part of the HIF1 transcriptional complex by virtue of a direct interaction with TRIP230. This effect is independent of other protein-protein interactions as the repressive effect of Rb was lost in cells over-expressing a transcriptionally competent mutant of TRIP230 lacking the Rb-interaction domain (Figure 6E and F) and in cells depleted of TRIP230 (Figure 3A). In addition, siRNA-mediated knock-down of Rb led to a concomitant increase of known HIF1 target genes in a hypoxia-dependent fashion in human breast MCF7 and in human prostate LNCaP cancer cell lines (Figure 1). Furthermore, we were able to record Rb over well-characterized HREs in the EPO and VEGF regulatory regions (Figure 2) suggesting that Rb may regulate expression of these genes at the transcriptional level.

The TRIP230 co-activator was cloned and characterized based on its ability to interact with the thyroid hormone receptor (TR) and enhance its transactivation function [13], [16], and by virtue of its ability to interact with Rb [16]. In this latter report, evidence was presented that supported a role for Rb as a transcriptional attenuator of thyroid hormone receptor function, likely mediated through the TRIP230 transcriptional co-activator. TRIP230 was later found to act as an essential co-activator for ARNT-dependent transcriptional activities, including hypoxia-inducible gene expression [12]. Importantly, we found that TRIP230 did not interact with HIF1α in a yeast two-hybrid assay (T. Beischlag, unpublished data). Our immuno-precipitation studies employing antibodies directed to TRIP230 demonstrate that endogenous ARNT and Rb interact with TRIP230 in MCF7 cells (Figure 6A) further supporting our hypothesis that Rb is part of a HIF1 transcriptional complex.

To further delineate the nature of this putative complex and to determine if ARNT, TRIP230 and Rb exist in a single complex, we eliminated other known protein-protein interaction interfaces within ARNT and used a GST pull-down strategy to affinity capture TRIP230 and Rb. The comprehensive analysis by GST pull-down performed by Elferink and colleagues failed to demonstrate a direct interaction with ARNT and Rb *in vitro* [30]. In addition, based on our previous studies characterizing the interaction between ARNT and TRIP230 [12], Partch and colleagues identified amino acids within the ARNT PAS-B domain that mediate the ARNT-TRIP230 interaction [31]. We designed a GST-ARNT-PAS-B fusion thus eliminating other protein interaction domains within ARNT. The ability of the ARNT-PAS-B region to pull-down Rb supports the existence of a multi-meric complex containing ARNT, TRIP230 and Rb (Figure 6B). Indeed, the PAS-B domain has emerged as a bona fide platform for the recruitment of multiple forms of transcriptional co-regulatory complexes [12], [15], [31], [33].

The HIF1 complex regulates the cell’s adaptive response to low oxygen mediating angiogenesis and alternative energy utilization via glucose metabolism. Hypoxia is also a hall-mark of solid tumors and has been implicated in tumor cell transformation. We found an exacerbated hypoxia-dependent accumulation of PLOD2 and CXCR4 mRNA in MCF7 and LNCaP cells depleted of Rb (Figure 1) and a concomitant increase in protein in MCF7 cells (Figure 4A).

This work represents the first link between the functional ablation of Rb in tumor cells and HIF1α-dependent invasion. These data support a hitherto unrecognized mode of action for Rb that is uncoupled from its canonical cell cycle/tumor suppressor function. Loss-of-function of Rb or genetic ablation of *RB1* has been implicated in advanced stages of brain cancers [34], [35], prostate cancer [36], [37], breast cancer, and lung cancer [20]. Furthermore, the loss of Rb and the activation of HIF1-regulated genes such as increased VEGF expression, microvascular hyperplasia and metastasis are traits that are common to progression in many solid tumors [19], however no direct link between the Rb and HIF pathways has been established. As a result, we were interested in further investigating the physiological connection between Rb function and hypoxia inducible gene expression, especially HIF1-regulated transcriptional programs involved in cancer cell transformation.

The observation that CXCR4 and PLOD2 protein (Figure 4A) and vimentin mRNA (Figure S2A) levels were elevated upon depletion of Rb from MCF7 cells led us to investigate the effects of Rb-loss on the invasive phenotype of MCF7 cells. CXCR4 expression is likely to be a key effector of the invasive phenotype observed in Figure 5 [38], [39]. CXCR4 promotes many key steps in epithelial to mesenchymal transition (EMT) and metastasis including detachment from neighboring cells, extra-vasation, metastatic colonization, angiogenesis and proliferation [40]. Furthermore, Gilkes and colleagues recently demonstrated that the metastatic marker PLOD2 is a hypoxia-inducible gene and is required for breast cancer metastasis to lymph and lung [41]. PLOD2 is an enzyme required for collagen production and plays a role in extracellular matrix re-modelling [42]. Under normoxic conditions MCF7 cells maintain an epithelial and non-invasive phenotype regardless of Rb status. Upon knock-down of Rb, hypoxia and CoCl_2_ treatment triggered invasion in nearly 3% of the cells seeded in the matrigel assay (Figure 5A, B and Figure S2C). This represents a change in the invasive potential of the MCF7 cells and it seems likely that loss of Rb contributes to HIF1-inducible tumor cell transformation. Our data suggests that Rb attenuates HIF1 function to ensure the appropriate levels of HIF1-target gene expression. Thus, we propose that loss of Rb or breakdown of this pathway primes cancer cells for metastatic transformation by allowing for the over-expression of pro-metastatic factors such as PLOD2 and CXCR4.

In addition, we were interested to see if known Rb-associated repressor complexes [26], [27] could be recorded over well-characterized HREs during hypoxia-driven transcription. ChIP analysis revealed the presence of Sin3a/b, HDAC1 and HDAC3 on the HIF-responsive regulatory regions of both the VEGF and EPO genes (Figure 2H). In light of the abundance of data suggesting that HDACs are required for HIF1-mediated transcription [43]–[45], we do not dismiss the theory that HDACs are essential for the initiation and maintenance of HIF1-regulated transcription. However, we are also cognizant that they may have pleiotropic effects and be recruited in a different fashion to attenuate the hypoxic response. Whether targeting these factors has any functional or therapeutic utility remains to be seen and will be the focus of future research efforts.

**Figure 7:**
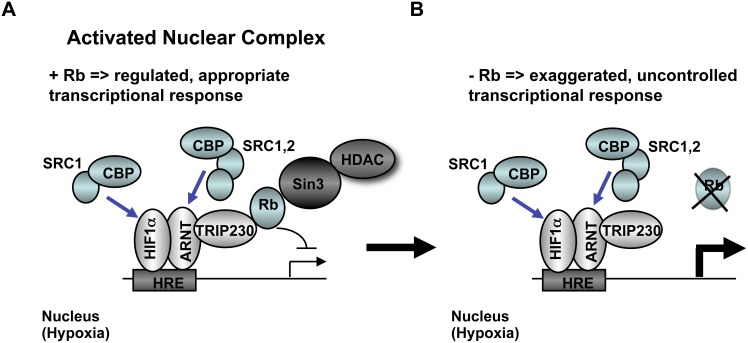
Illustration describing the transcriptional regulation of HIF-target genes in cells either expressing or lacking Rb. (**A**) In cells that are Rb-positive, the full transcriptional activation capacity of the HIF1-TRIP230 complex is repressed or muted resulting in regulated expression of HIF1 target genes. (**B**) In cells lacking Rb, gene expression mediated by HIF1 becomes uncontrolled.

The hypothesis that ARNT, TRIP230 and Rb act in concert to regulate hypoxia-inducible gene transcription is supported by several lines of investigation. First, our data derived from the sequential chromatin immuno-purifications using ARNT, HIF1α, TRIP230 and Rb affinity purified antibodies (Figure 2D and E) suggest that all 4 proteins are present at HIF1-regulatory elements at the same time. Second, a transcriptionally competent mutant of TRIP230 (TRIP230ΔRb) overcomes the repressive effect of Rb over-expression on hypoxia-inducible transcription in Rb-negative Hepa1 cells (Figure 6E). Third, TRIP230ΔRb was more efficacious in co-activating hypoxia-inducible transcription in Rb-positive MCF7 cells (Figure 6F). Finally, loss of Rb did not exacerbate the hypoxic response in cells depleted of TRIP230 (Figure 3A). Taken together, there is strong evidence that Rb is a negative regulator of the TRIP230-HIF1 complex and that loss of Rb unmasks the full co-activation potential of TRIP230 (Figure 7).

While we have not ruled out the involvement of hypo-phosphorylated Rb in HIF1 function, the presence of hyper-phosphorylated Rb at HIF regulatory elements and after GST pull-down by the ARNT-PAS-B domain (Figure 6C and D) supports the observations of other investigators [16], [17]. Since there was no appreciable difference in the ability of the GST-ARNT-PAS-B moiety to pull-down Rb from either normoxic or hypoxic extracts, it seems as if neither HIF1α nor HIF2α is essential for this interaction. Additionally, it seems clear that the modulatory effect of Rb on HIF function is uncoupled from E2F as evidenced by the unaltered transcriptional response observed after knock-down of DP1 (Figure 3C). Furthermore, the abundance of phospho-Rb (serine^780^ and serine^807/811^) (Figure 4B) indicates that Rb is in a permissive state for uncoupling from E2F [46]. Thus, our data suggest that Rb plays an essential role in regulating the amplitude of the hypoxic response for genes regulating both angiogenesis and metastasis and that loss of Rb leads to exacerbated expression of HIF1α target genes that regulate tumor progression.

Targeting angiogenesis has been an attractive strategy for combating cancer [47] however, there are caveats to anti-angiogenic therapies. Avastin, a monoclonal antibody to VEGF failed to extend survival rates in patients suffering from breast cancer [48], and is of limited benefit in other types of cancer [49]. Furthermore, there is experimental evidence that anti-angiogenic drugs exacerbate tumor progression [50]. Therefore, a novel approach to combating tumor progression may involve specifically targeting the HIF1α/β-TRIP230-Rb complex, that regulates both angiogenic and cell invasion programs [51]. We have demonstrated that Rb represses or attenuates the co-activation function of TRIP230 and thereby regulates the transcriptional response to hypoxia. In addition, this work demonstrates that Rb plays a hitherto unidentified role in tumor suppression by virtue of effects on HIF1α/β that is distinct from its classic tumor suppressor role mediated through repression of E2F transcription factors. These data provide the first direct link between the loss of Rb and HIF1-regulated pro-metastatic and pro-angiogenic processes. Therefore, targeting this pathway could yield novel therapies to better combat solid tumor progression and metastasis.

## Materials and Methods

### Cell Culture, Transient Transfections, Luciferase Assays and RNA Interference

Hepa1c1c7 and HEK293T cells (ATCC) were maintained in Dulbecco’s Modified Eagle’s Medium (DMEM; BioWhittaker, Lonza) with 10% fetal bovine serum (FBS; HyClone, Perbio, Thermo Fisher Scientific Inc.). MCF7 cells were maintained under similar conditions but were supplemented with 100 units/ml potassium penicillin-100 µg/ml streptomycin sulphate (BioWhittaker, Lonza), and 4.5 g/L glucose and 4.5 g/L L-glutamine at 37°C, 20% O_2_, and 5% CO_2_. LNCaP cells were maintained in RPMI 1640 medium with L-Glutamine (BioWhittaker, Lonza) supplemented with 10% FBS and 100 units/ml potassium penicillin-100 µg/ml streptomycin sulphate. Transient transfections and luciferase assays were performed as described previously [12]. Rb, and TRIP230 wild-type and TRIP230 mutant expression plasmids were generously provided by Dr. Y. Chen (Univ. of Texas, San Antonio) and were described previously [16].

MCF7 or LNCaP cells were transfected with either scrambled (SCX) siRNA (DS Scrambled negative control siRNA, Integrated DNA Technologies Inc.), Rb siRNAs (Integrated DNA Technologies Inc., Cat. No. HSC.RNAI.N000321.9.1, HSC.RNAI.N000217.9.2; siRb 1, and siRb 2, respectively), ARNT siRNA (Integrated DNA Technologies Inc., Cat. No. HSC.RNAI.N178426.11.3), TRIP230 siRNA (Integrated DNA Technologies Inc., Cat. No. HSC.RNAI.N004239.12.1) or DP1 siRNA (Integrated DNA Technologies Inc., Cat. No. HSC.RNAI.N007111.11.1). MCF7 cells were transfected with 10–15 nM siRNA using 0.3% (v/v) Lipofectamine RNAiMAX (Invitrogen Inc) according to manufacturer’s protocol. The cells were allowed to incubate in transfection mix for 6 h at 37°C, 20% O_2_, and 5% CO_2_ after which the transfection mix was removed and replaced with complete media.

### Antibodies

The mouse anti-TRIP230 IgG was kindly provided by Dr. Y. Chen (University of Texas, San Antonio). Anti-Rb (rabbit polyclonal, Santa Cruz Biotechnology Inc., SC-7905), anti-phospho-Rb (Ser^780^) (Cell Signaling, 9307), anti-phospho-Rb (Ser^807/811^) (Cell Signaling, 9308), anti-PLOD2 (mouse polyclonal, Abnova, H00005352-B01P), anti-HIF1α (rabbit polyclonal, Santa Cruz Biotechnology Inc., SC-10790), anti-DP1 (rabbit polyclonal, Santa Cruz Biotechnology Inc., SC-610), anti-ARNT (goat polyclonal, Santa Cruz Biotechnology Inc., SC-8076), anti-CXCR4 (rabbit polyclonal, Abcam Inc., ab2074), anti-α-tubulin (mouse monoclonal, Santa Cruz Biotechnology Inc., SC-8035), anti- HIF2α (mouse monoclonal, Santa Cruz Biotechnology Inc., SC-46691), goat anti-rabbit IgG-HRP (Santa Cruz Biotechnology Inc., SC-2004), goat anti-mouse IgG-HRP (Santa Cruz Biotechnology Inc., SC-2005), donkey anti-goat IgG-HRP (Santa Cruz Biotechnology Inc., SC-2020).

### Quantitative Real-Time PCR

For quantitative real-time PCR (RT-PCR) experiments, MCF7 or LNCaP cells were incubated under hypoxic conditions (1% O_2_) for 24 h in a humidified CO_2_ incubator. The mRNA levels of VEGF, EPO, CXCR4, vimentin, PLOD2, RB1, and 36B4 were determined using quantitative real-time PCR. The primer pairs for VEGF EPO and 36B4 were described previously [4]. The other primer pairs used were; CXCR4: 5′-CAGTGGCCGACCTCCTCTT-3′ and 5′-GGACTGCCTTGCATAGGAAGTT-3′; RB1: 5′- CATCGAATCATGGAATCCCT-3′ and 5′- GGAAGATTAAGAGGACAAGC- 3′; PLOD2 5′-GCGTTCTCTTCGTCCTCATC-3′ and 5′-GTGTGAGTCTCCCAGGATGC-3′, and; Vimentin: 5′-TTCCAAACTTTTCCTCCCTGAACC-3′ and 5′-TCAAGGTCATCGTGATGCTGAG-3′; Total RNA was isolated using TRI reagent (Sigma, Cat. No. T9424-200ML) according to the manufacturer’s protocol. Reverse transcription was performed using High Capacity cDNA Reverse Transcription Kit (Applied Biosystems, Part No.4368814) according to the manufacturer’s protocol. A total of 2–4 µg of RNA was used in a 20 µL reaction amplified by cycling between 25°C for 5 min, 37°C for 120 min, and 85°C for 5 min (Veriti 96 Well Thermal Cycler, Applied Biosystems). From each experiment, a sample that was both transfected with Rb-specific siRNA and pre-conditioned with hypoxia was used to generate a relative standard curve in which the sample was diluted 1∶10 in five serial dilutions resulting in dilutions of 1∶10, 1∶100, 1∶1,000, 1∶10,000, and 1∶100,000 whereas the samples were diluted 1∶30; the analysis was done using StepOnePlus System (Applied Biosystems).

### Chromatin Immuno-precipitation Assays

Chromatin immuno-precipitations (ChIPs), and sequential ChIP assays were performed as described previously [12], [52]. Oligonucleotide sequences for PCR amplification of human VEGF and EPO regulatory regions were as described previously [12]. All antibodies were supplied by Santa Cruz Biotechnology Inc. or as described above.

### Immuno-blotting

Protein analysis was performed by immuno-blotting as described previously [53]. Briefly, MCF7 cells were incubated under hypoxic conditions (1% O_2_) for either 48 h or 96 h. Cells were harvested and the protein concentration estimated by the Bradford assay. Equal amounts of proteins from the samples were resolved on a SDS-acrylamide gel then transferred to polyvinylidene fluoride (PVDF) membrane. Membranes were incubated with diluted primary antibodies in 5% w/v skim milk powder, 1X TBS, 0.1% Tween-20 at 4°C with gentle shaking, overnight. The detection was done using horseradish peroxidase conjugated anti-mouse or anti-rabbit IgG (Santa Cruz Biotechnology Inc.) and ECL Prime detection kit (GE Healthcare).

### Matrigel Invasion and Cell Proliferation Assays

MCF7 cells were transfected with scrambled siRNA or *Rb* siRNA as described above. Twenty-four hours after siRNA transfection, the cells were washed, trypsinized, and re-suspended in culture medium, and subjected to invasion assay using BD BioCoat Matrigel Invasion Chamber (BD Sciences, Cat. No. 354480) according to the manufacturer’s protocol. Briefly, the suspended chambers were rehydrated in warm bicarbonate-based medium for 2 h. MCF7 cells were reverse transfected according to manufacturer’s protocols and seeded into invasion chambers in DMEM without FBS at a density of 10,000 cells/chamber. Chambers were placed in 24-well plates with chemo-attractant (complete medium containing 10% FBS) in the well. The plates were incubated in normoxic (20% O_2_) or hypoxic conditions (1% O_2_) at 37°C for 24 h or treated with vehicle or 100 µM CoCl_2_ for 24 h. Before mounting the invasion membrane to microscope slides, the non-invading cells were removed by cotton swab and invading cells in the membrane were fixed with 100% methanol and stained with 1% toluidine blue. All the cells in the invasion membrane were counted using light microscopy at 10–40× magnification. Assays were performed in triplicate and each membrane was counted three times.

MCF7 cells grown to 75–80% confluence were transfected with either Rb or scrambled negative control siRNA (SCX). Media was changed after eight hours. Twenty-four h post-transfection, cells were washed 2 times with PBS, trypsinized and seeded into 6-well plates at 10,000 cells/well. Twenty-four h after plating, CoCl_2_ was added directly to half the wells to a final concentration of 100 µM. Cells were counted at; 0 (control), 6, 12, 24, 36, 48 and 72 h following CoCl_2_ administration. Determinations were performed in triplicate and each sample was counted three times.

### Flow Cytometry and Sub-G1 Status

Cell cycle status was determined by propidium iodide (PI) staining and flow cytometry. MCF7 cells treated with a scrambled negative control or with Rb knocked-down, were treated with hypoxia or left at normoxic conditions for 36-hours and then harvested using trypsin. Biological triplicates of 5×10^5^ cells were fixed in 70% ethanol on ice for 15 minutes and then cells were centrifuged for 3 minutes at 1500 rpm to remove the ethanol and incubated in 0.5 ml of propidium iodide staining solution (50 µg/mL PI, 0.05% Triton X-100, 0.1 mg/mL RNase A, in PBS) for 40 min at 37°C. Following staining, cells were washed with PBS then run on a BD FACSCanto II flow cytometer (488 nm excitation, 617 emission, 375 volts, PI) where 20,000 events were collected. The percentage of cells in each stage of the cell cycle was determined using FlowJo analysis software based on the PI staining profile of FSC/SSC-gated population.

### Co-immuno-precipitation and Glutathione-s-transferase Pull-down Assays

Immuno-precipitation of complexes from MCF7 cell nuclear extracts was performed essentially as described previously [15], with minor modifications. MCF7 cells were maintained in 1% O_2_ for 6 h in a humidified CO_2_ incubator and nuclear extracts were prepared following our established protocol [54]. Approximately, 1 mg of nuclear extract was incubated with 5 µg of either affinity purified anti-TRIP230 or control mouse IgG at 4°C for 90 min on a spinning wheel. Approximately, 50 µl of pre-cleared Protein-G sepharose beads were added to each mixture and incubated for a further 90 min. Samples were washed vigorously and fractionated by SDS-PAGE. Precipitates were transferred to nylon membrane for immuno-blotting and blots were probed with antibodies to TRIP230, ARNT and Rb.

Glutathione-*s*-transferase (GST) pull-down assays were performed as described previously with minor modifications [15]. In order to construct the GST-ARNT-PAS-B fusion moiety, a mouse ARNT cDNA encoding amino acids 344–479 was PCR-amplified and cloned into pGEX-5X-1 using Bam H1 and XhoI. Approximately 30 µl of GST or GST-ARNT-PAS-B beads were incubated with 250 µg of nuclear extract at 4°C for 90 min on a spinning wheel, washed and eluted as described previously [15]. Eluted samples were fractionated by SDS-PAGE, transferred to PVDF membrane and analyzed by immuno-blot for the presence of TRIP230, Rb or phospho-Rb (Ser^780^).

### Statistical Analysis

Statistical analyses were performed using GraphPad Prism 4.0. For multiple comparisons (i.e. siRNA experiments) statistical significance was determined using a 2-way ANOVA with Tukey’s Multiple Comparison test. Values are presented as means ± standard deviation (S.D.). A P value <0.05 was considered to be significant.

## Supporting Information

Figure S1 **Complete digital images of (A) immuno-blots depicted in manuscript** **Figures 4A** **, (B) 6A, (C and D) 6B (E) 6H and (F) 2C.** When necessary, blots were cut into strips at encompassing the appropriate molecular weights to that different proteins of interest of different molecular weights could be analyzed from the same sample and expt. For GST pull-down experiments, the entire blot cut into two sections is shown. However, we used the pull-down of TRIP230 from another identical experiment (Figure S1B) as a representative blot because the signal was stronger. In some cases, brightness and contrast of the images have been altered (*) in order for the boundary of the cut blots or for the molecular weight markers to be clearly visible.
(TIF)

Figure S2 **(A)** Relative mRNA levels of vimentin under similar conditions described in Figure 1. Open bars represent normoxia (20% O_2_) and closed (grey) bars represent hypoxia (1% O_2_). *p<0.05. **(B)** Titration of Rb expression vector into Hepa1C1C7 cells. Increasing amounts of Rb expression vector was co-transfected with pCMV-TRIP230 and an HRE-driven luciferase vector (see Materials and Methods and Figure 6 legend). **(C)** Numerical representation of relative invasion of matrigel-embedded MCF7 cells after treatment with SCX or siRb and treatment with the HIF1 activator, CoCl_2_ (100 µM).
(TIF)
