# Supplementary figures and images for: A TRIP230-Retinoblastoma Protein Complex Regulates Hypoxia-Inducible Factor-1α-Mediated Transcription and Cancer Cell Invasion
Source: PLoS One. 2014 Jun 11;9(6):e99214. doi: 10.1371/journal.pone.0099214 (PMC4053355; doi:10.1371/journal.pone.0099214)

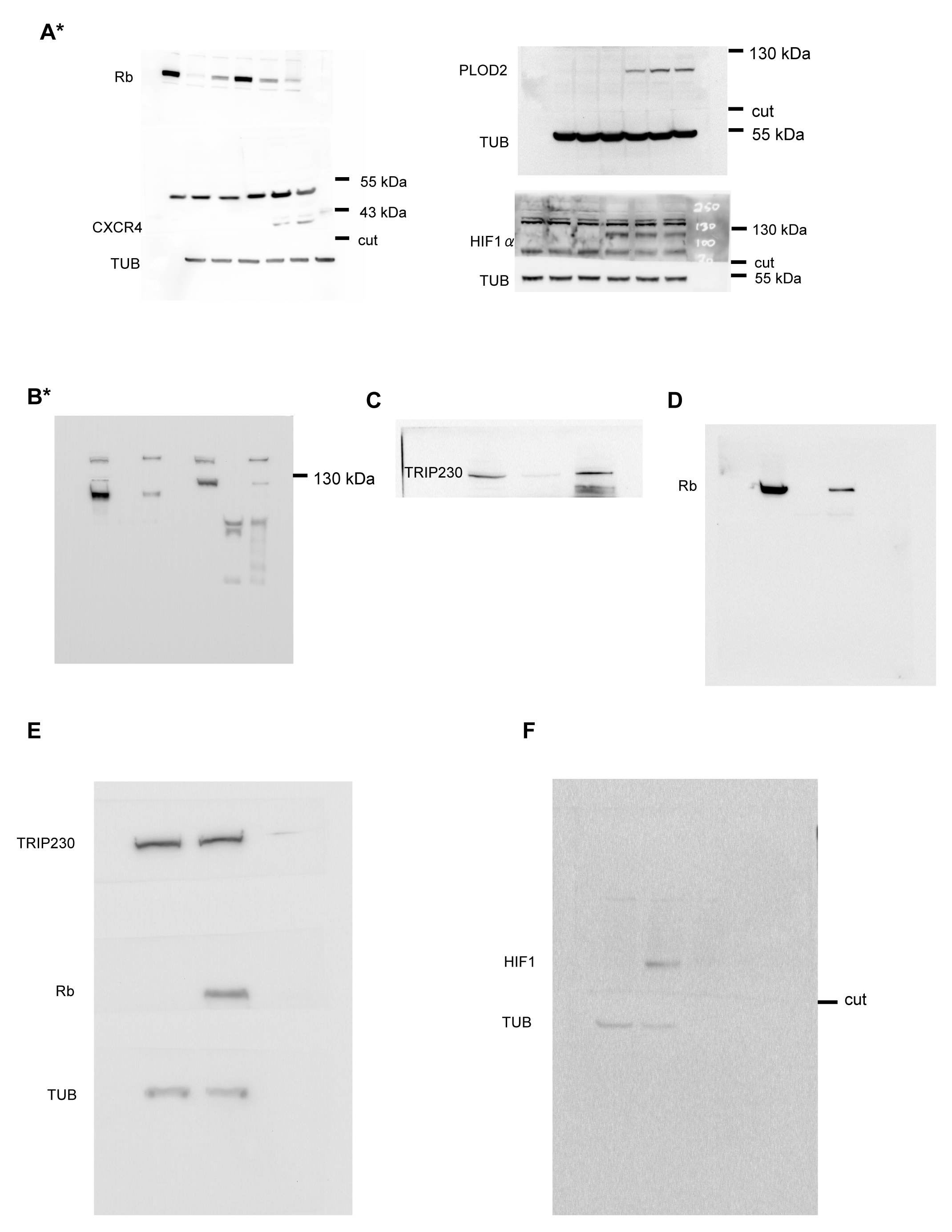

Supplement: Figure S1 — Complete digital images of (A) immuno-blots depicted in manuscript Figures 4A , (B) 6A, (C and D) 6B (E) 6H and (F) 2C. When necessary, blots were cut into strips at encompassing the appropriate molecular weights to that different proteins of interest of different molecular weights could be analyzed from the same sample and expt. For GST pull-down experiments, the entire blot cut into two sections is shown. However, we used the pull-down of TRIP230 from another identical experiment (Figure S1B) as a representative blot because the signal was stronger. In some cases, brightness and contrast of the images have been altered (*) in order for the boundary of the cut blots or for the molecular weight markers to be clearly visible. (TIF) [file pone.0099214.s001.tif]

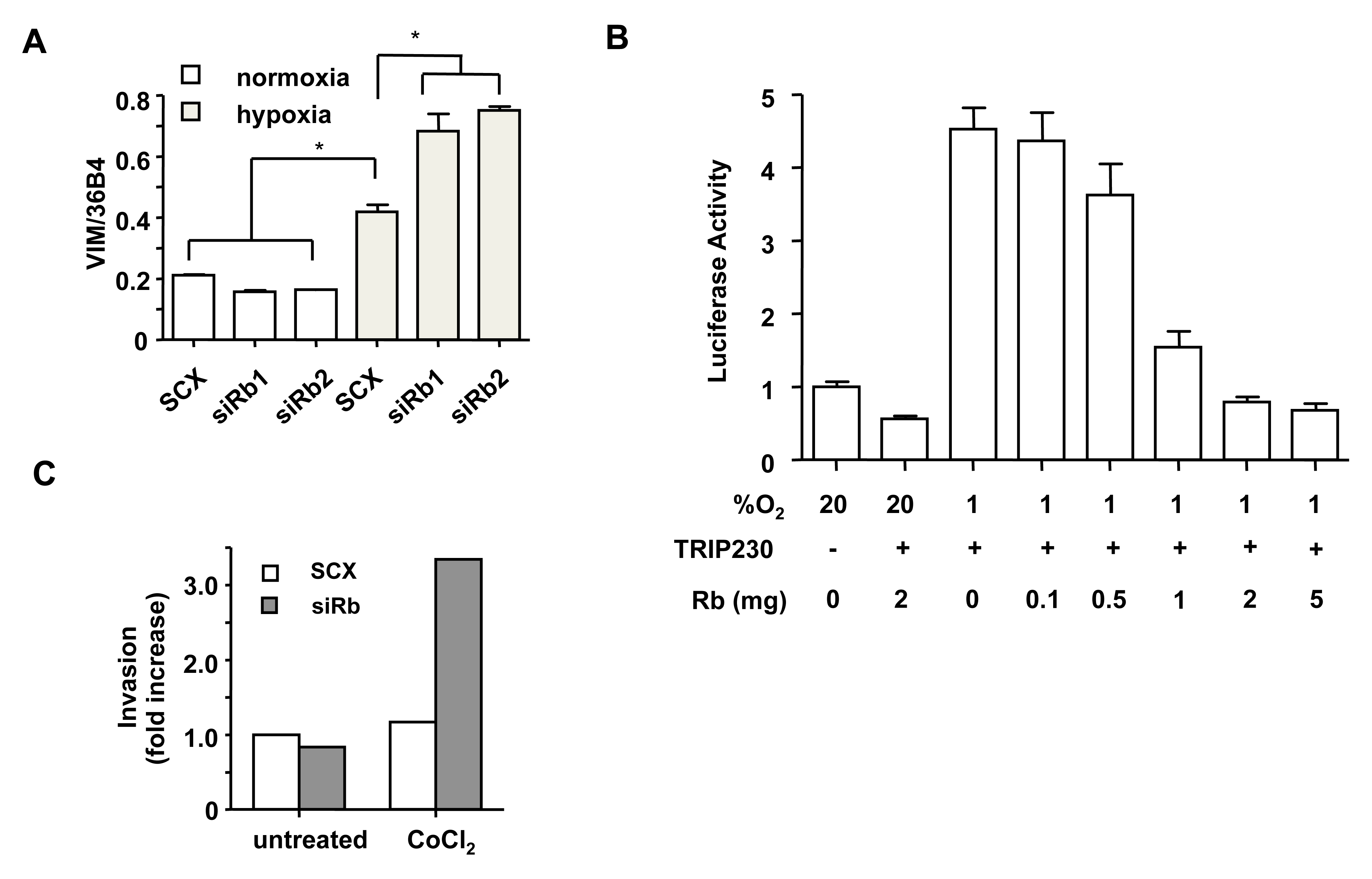

Supplement: Figure S2 — (A) Relative mRNA levels of vimentin under similar conditions described in Figure 1. Open bars represent normoxia (20% O2) and closed (grey) bars represent hypoxia (1% O2). *p<0.05. (B) Titration of Rb expression vector into Hepa1C1C7 cells. Increasing amounts of Rb expression vector was co-transfected with pCMV-TRIP230 and an HRE-driven luciferase vector (see Materials and Methods and Figure 6 legend). (C) Numerical representation of relative invasion of matrigel-embedded MCF7 cells after treatment with SCX or siRb and treatment with the HIF1 activator, CoCl2 (100 µM). (TIF) [file pone.0099214.s002.tif]
